# Improved DNA Extraction for Dairy and Blood Products: A Comparative Evaluation of Yield, Purity, and PCR Compatibility

**DOI:** 10.3390/foods15101790

**Published:** 2026-05-18

**Authors:** Xiaorong Xu, Jie Fang, Lingyan Mao, Yingying Wu, Hai Cheng, Jinru Lin, Liyu Shi, Jiali Xing, Xiaohu Luo

**Affiliations:** 1 Key Laboratory of Detection and Control of Hazardous Materials in Food, China General Chamber of Commerce, Ningbo Academy of Product and Food Quality Inspection (Ningbo Fibre Inspection Institute), Ningbo 315048, China; 1711091100@mail.nbu.edu.cn (X.X.);; 2 College of Biological and Environmental Sciences, Zhejiang Wanli University, Ningbo 315199, China; 3 College of Food Science and Engineering Sciences, Ningbo University, Ningbo 315211, China

**Keywords:** DNA extraction, dairy products, blood products, food authenticity detection

## Abstract

DNA must be efficiently extracted from samples to accurately test the authenticity of food, particularly from processed matrices in which DNA integrity may be compromised. We systematically evaluated the efficiency of extracting DNA from dairy and blood products by four methods, namely SDS-CTAB, SDS-isopropanol precipitation, guanidine isothiocyanate magnetic beads, and a commercial kit. The guanidine isothiocyanate-magnetic bead method yields high quantities and purity of DNA; for example, the yield obtained from chicken blood samples was 318.34 ± 4.77 ng/µL, with an A260/A280 ratio ranging from 1.8 to 2.0. The processing time of this method was compared with the DNA Extraction Kit shorter by 40% and unlike methods such as the SDS-CTAB protocol, does not require the use of toxic reagents such as phenol or chloroform, meeting green chemistry requirements. Among the dairy and blood samples tested, it enables the extraction of DNA in quantities comparable to those obtained using commercial kits; moreover, the DNA yield achieved is 20–30% higher than that of these kits. Furthermore, this method is free from the limitations associated with protein contamination and amplification instability often encountered in protocols such as the CTAB-SDS and SDS-isopropanol methods. The magnetic bead approach was adaptable for complex matrices and demonstrated strong tolerance to coexisting contaminants, thereby improving extraction performance in challenging food samples. The magnetic bead surface functionalization and buffer systems could be improved to further increase their versatility. This method enables reliable DNA extraction and advanced technical support for DNA analysis.

## 1. Introduction

The global food industry has vastly transformed in response to stricter regulations and consumer expectations regarding food quality and safety [1]. Dairy products, particularly milk, have become increasingly larger components of the global daily diet as part of this transformation. Infant formula is now widely used in many countries as an alternative to breastfeeding, and dairy consumption plays a crucial role in supporting adolescent growth and development [2,3]. From 2000 to 2015, the per capita consumption of dairy products in Southeast Asia increased from 42.3 kg to 53.7 kg [4]. In addition, the intake of milk and dairy products by adult residents in China was 10.2 kg per year on average in 2018 and increased to 41.3 kg per year in 2023 according to the China Health and Nutrition Survey [5]. Additionally, the economic importance of blood-based food products has increased worldwide due to their high nutritional value. These blood-based products are strong sources of high-quality proteins, essential minerals (notably iron and zinc), and vital vitamins, such as B12 and folate [6]. The duck blood market in China is large, with an estimated value of approximately 500 million dollars. Industry reports indicate that the duck blood market has achieved a multimillion-dollar status globally, and it is expected to have an annual growth rate of 6.2% [7,8].

Food fraud remains a considerable challenge in the dairy and blood-based food sectors [9]. Economically motivated adulteration has become increasingly prevalent, with lower-value animal components frequently being used as substitutes for premium products [10]. Examples include the adulteration of duck blood with pig blood, the substitution of deer blood with lower-value alternatives [11], and mixing goat or camel milk with cow milk for sale as higher-value products [12]. These fraudulent practices not only undermine consumer trust but also pose health risks, particularly for individuals with specific allergies or dietary restrictions [10].

Various analytical techniques have been used to combat food adulteration practices, such as molecular-biology-based assays, electrophoretic techniques, and chromatographic methods [13,14,15]. These approaches rely on the detection of species-specific proteins or metabolic signatures. The DNA-based methods, such as polymerase chain reaction (PCR), are useful because of their high specificity and sensitivity for identifying species [16]. However, the effectiveness of PCR is hindered by the presence of inhibitory substances, such as polysaccharides, lipids, and other compounds [17], which are commonly found in complex food matrices. These inhibitors cannot be efficiently removed using conventional DNA extraction methods, which compromises the reliability of downstream molecular analyses. Therefore, efficient DNA extraction from diverse food matrices serves as a fundamental prerequisite for downstream analytical applications [18].

Various DNA extraction protocols have been developed to address these challenges experienced with PCR, such as those based on cetyltrimethylammonium bromide (CTAB) and sodium dodecyl sulfate (SDS) [19]. Among these, the modified CTAB method and magnetic bead-based techniques are the most widely applied and have been developed into kits. However, the performance of these methods considerably varies depending on the specific food matrix being analyzed, and the cost of using the kits is relatively high [20]. Therefore, there is a growing need to develop more cost-effective, reliable, and broadly applicable DNA extraction methods that yield high-purity DNA across diverse food matrices. The aim in this study was to address this issue by systematically evaluating alternative DNA extraction methodologies with two primary objectives: to compare the DNA purity and quality obtained from various blood and dairy products and to improve extraction protocols tailored to different food matrices. The improved DNA extraction method developed in this study provides a robust foundation for downstream applications such as PCR and qPCR.

## 2. Materials and Methods

### 2.1. Reagents and Samples

Sodium chloride (NaCl), sodium hydroxide (NaOH), hydrochloric acid (HCl), tris (hydroxymethyl) aminomethane hydrochloride (Tris-HCl), acetone, trichloromethane (CHCl_3_), isoamyl alcohol, ammonium sulfate (NH_4_Cl), ethylenediaminetetraacetic acid disodium salt (EDTA-Na_2_), ethylenediaminetetraacetic acid (EDTA), sodium dodecyl sulfate (SDS), cetyltrimethylammonium bromide (CTAB), isopropanol (IPA), 20 mg/mL proteinase K, β-mercaptoethanol, and Triton X-100 were purchased from Adamas (Shanghai, China). Phosphate-buffered saline (PBS) without calcium and magnesium ions was purchased from Gibco (Beijing, China). Ethanol absolute, potassium acetate (KOAc), and guanidine isothiocyanate (GITC) were obtained from Greagent (Shanghai, China). Polyethylene glycol sorbitan monolaurate (Tween 20) from Beyotime (Shanghai, China). EasyPure Food and Fodder SecurityGenomic DNA Kit was purchased from Beijing TransGen Biotech Co., Ltd. (Beijing, China); TAE buffer was obtained from Aladdin (Shanghai, China). The magnetic beads (50 mg/mL) and primers (100 uM/μL) were synthesized by Ningbo Hanjing Biotechnology Co., Ltd. (Ningbo, China); Polyvinylpyrrplidone (PVP-40, MW~40,000) form Nanjing Reagent (Nanjing, China); N-Lauroylsarcosine form Aladdin (Ningbo, China). The 5% BSA blocking fluid is from Solarbio (Beijing, China). Premix Taq^TM^ (TaKaRa Taq^TM^ Version 2.0 plus dye), Premix Ex Taq probe-based qPCR detection reagent was purchased from TaKaRa (Dalian, China).

High-temperature-sterilized milk, yogurt, goat milk powder, milk slices, milk powder, chicken blood, duck blood, pig blood, high-calcium milk, whey protein powder, blood sausage, fresh duck blood, lard, and starch were purchased from an online shopping platform and local supermarkets in Zhejiang, China.

### 2.2. Equipment

The pH of the experimental solutions was measured using a calibrated pH meter (INASE, PHS-3C, Shanghai, China). An electronic analytical balance (Setra Systems, BL-2000F, Tianjin, China) with a precision of 0.001 g was utilized for weighing the samples. A vortex shaker (IKA, VORTEX 3, Guangzhou, China) was employed to ensure thorough mixing of the solutions. Homogenization of the samples was performed using a bio-homogenizer (Bertin Pharma, Precellys Evolution, Montigny-le-Bretonneux, France) to achieve uniform dispersion. A metal water bath (Thermo Fisher Scientific, Thermo Scientific™, Waltham, MA, USA) was used to maintain the required temperature for cell membrane lysis and DNA release. DNA centrifugation was conducted using a refrigerated benchtop centrifuge (Thermo Fisher Scientific, Micro 17R, Waltham, MA, USA). The magnetic rack was used for the adsorption, separation, and purification of magnetic beads in solutions (BaseLine, Affimag 51512, Tianjin, China).

The success of DNA extraction was evaluated by PCR (Thermo Fisher Scientific, ProFlex™ Base, Waltham, MA, USA) and agarose gel electrophoresis (Thermo Fisher Scientific, EPS-300X, Waltham, MA, USA) to assess the integrity and amplification efficiency of the extracted DNA. DNA concentration and purity were quantified using a micro-volume ultraviolet spectrophotometer (MAESTRO, MN-913, Hsinchu, Taiwan). The electrophoretic results were visualized and documented with a gel imaging system (UVP, GelDoc-It2, Bielefeld, Germany).

### 2.3. Sample Preparation

For the preparation of dairy product samples, 1 mL of the dairy product was mixed with twice its volume of pre-cooled acetone, vortexed thoroughly, and incubated at −20 °C for 30 min. The mixture was then centrifuged at 12,000× *g* for 10 min at 4 °C. After centrifugation, the supernatant was centrifuged at 12,000× *g* for 10 min at 4 °C. After centrifugation, the supernatant was carefully decanted, and the upper fat layer and middle emulsion layers were completely removed. The sediment was then resuspended in 1.5 mL of sterile PBS (pH 7.4) and centrifuged at 12,000× *g* for 10 min at 4 °C. The supernatant was discarded, and the washing step was repeated 2–3 times. The precipitate was kept and stored at −20 °C. In all subsequent methods, the sample dosage shall is mainly based on this precipitate. Samples were taken from the same batch and three sets of parallel tests were conducted to verify the repeatability of the DNA extraction methods [21,22]. For solid samples, such as ground milk slices and milk powder, 200 mg of each product was weighed and was placed into 5 mL centrifuge tubes; they were then processed following the same steps as for dairy products.

The blood product samples were prepared as follows. A 200 μL aliquot of each sample was mixed with 1 mL of ice-cold erythrocyte lysis buffer (155 mM NH_4_Cl, 10 mM KHCO_3_, 0.1 mM EDTA-Na_2_, pH 7.4), vortexed thoroughly, and incubated on ice for 10 min. The mixture was then centrifuged at 3000× *g* for 10 min at 4 °C, and the supernatant was discarded. The sediment was resuspended in 1.5 mL of sterile PBS (pH 7.4); this process was repeated 1–2 times. The precipitate was kept and stored at −20 °C. In all subsequent methods, the sample dosage is mainly based on this precipitate. Samples were taken from the same batch and three sets of parallel tests were conducted to verify the repeatability of the DNA extraction methods [23]. Solid blood products (coagulated duck blood, pig blood, etc.) were prepared by weighing 200 mg of each product, which was then ground using a food processor. The same processing steps were followed for solid blood products. Extraction was performed using a guanidinium isothiocyanate-based magnetic bead method, with 1% (*w*/*v*) N-lauroylsarcosine added to the erythrocyte lysis buffer.

### 2.4. Improve of DNA Extraction Method Parameters

Based on the literature review and comparison of relevant methods, the following four methods were selected for extracting DNA from dairy and blood products: SDS-isopropanol precipitation (SDS-IPA precipitation), guanidine isothiocyanate-magnetic bead extraction (GITC-Magnetic bead), SDS-CTAB, and a commercial DNA Extraction kit. To verify the differences in extraction efficiency among various methods and minimize the influence of proteinase K dosage and incubation time, milk and pig blood were chosen as the samples in the preliminary experiments. While keeping all other procedures constant, we adjusted the proteinase K dosage and incubation time for each of the four methods to reduce variability caused by these two factors.

### 2.5. Screening DNA Extraction Methods

#### 2.5.1. SDS-Isopropanol Precipitation Method

The DNA extraction protocol was modified from the SDS-IPA method originally developed by Huang et al. [24]. We introduced modifications and applied them to the extraction of DNA from dairy and blood products. The pretreated DNA solution was combined with 600 μL of Buffer 1 (2% SDS, 100 mM Tris-HCl, 0.5% Triton X-100, 1% PVP-40, 50 mM EDTA, 1.4 M NaCl, 0.5% β-mercaptoethanol (add as needed; pH 8.0) and 10 μL of proteinase K (10 mg/mL)). The mixture was incubated at 65 °C for 1 h [25] and then allowed to cool to 20 °C. Subsequently, 400 μL of the reaction mixture was combined with 165 μL of 5 M KOAc solution (pH 4.8), vortexed thoroughly, and incubated on ice for 10 min. Following centrifugation at 12,000× *g* for 5 min to precipitate protein-SDS complexes, 300 μL of the supernatant was carefully transferred to a new tube. We added 600 μL of ice-cold isopropanol to the supernatant, and the solution was thoroughly mixed and incubated at 20 °C for 10 min, which was mixed by inversion and centrifuged at 12,000× *g* for 8 min to precipitate the DNA. The resulting supernatant was discarded, and the DNA was washed twice with 500 μL of 70% ethanol at 12,000× *g* for 1 min to remove any residual salts. The pellet was completely air-dried at 20 °C for 20 min to allow the ethanol to evaporate; the DNA was resuspended in 50 μL of TE buffer (10 mM Tris-HCl and 1 mM EDTA; pH 8.0) and thoroughly vortexed to ensure complete dissolution and stored at −20 °C until use.

#### 2.5.2. SDS-CTAB Method

DNA extraction was performed using an SDS-based method adapted from Xia et al. [26], with minor modifications to improve yield and purity for dairy and blood product samples. A total of 500 μL of SDS extraction buffer (2% SDS, 100 mM Tris-HCl, 50 mM EDTA, 1.0 M NaCl, 0.5% β-mercaptoethanol (add as needed; pH 8.0) and 10 μL of proteinase K (10 mg/mL)) were added to the sample. The mixture was thoroughly mixed and incubated at 65 °C for 60 min. The reaction mixture was combined with 165 μL of 5 M potassium acetate solution (pH 4.8), mixed thoroughly, and incubated in an ice water bath for 10 min. The sample was centrifuged at 12,000× *g* for 10 min, and 300 μL of the supernatant was collected. Then, 300 μL CTAB buffer (2% CTAB, 100 mM Tris-HCl, 1% PVP-40, 20 mM EDTA, 1.4 M NaCl; pH 8.0) was added to the sample, which were thoroughly mixed and incubated at 65 °C for 15 min. An equal volume of chloroform: isoamyl alcohol (24:1, *v*/*v*) was added to the sample, which was mixed and centrifuged at 12,000× *g* for 10 min, after centrifugation, the supernatant was removed. A total of 600 μL of ice-cold isopropanol was then added to the sample, which were mixed well and placed in a freezer at −20 °C for 10 min. The sample was centrifuged at 12,000× *g* for 30 min, the supernatant was discarded, and the DNA was retained. The resulting pellet was washed twice with 70% ethanol. The DNA was air-dried at 20 °C for 30 min, dissolved in 50 μL of TE buffer, and stored at −20 °C until use [27].

#### 2.5.3. Guanidine Isothiocyanate-Magnetic Bead Method

The magnetic bead method for DNA extraction is based on the dosage of DNA reagents in plant protein beverages and the research on SARS-CoV-2 BA virus mutations [28,29], with subsequent modifications and enhancements to improve protocol efficacy. We added 10 μL of proteinase K (10 mg/mL) and 200 μL of cell lysis buffer (4 M guanidine isothiocyanate, 40 mM EDTA, 50 mM Tris-HCl, 1% PVP-40, and 2% Triton X-100; pH 6.8) to the samples. The mixture was incubated at 65 °C for 10 min, then heated at 80 °C for 5 min to inactivate the enzyme and prevent DNA degradation. After incubation was complete, 200 μL of DNA adsorption buffer (40% isopropanol, 2.5 M NaCl, 20 mM Tris-HCl; pH 6.5), and 20 μL of magnetic bead suspension was added to the solution. The solution was thoroughly mixed using a vortex mixer and allowed to react at 20 °C for 5 min. The centrifuge tube was placed on a magnetic rack for 1 min, after which the solution had settled, and the supernatant was removed. Next, 500 μL of wash buffer I (3 M guanidine isothiocyanate, 20 mM Tris-HCl, 0.1% Tween-20; pH 6.5) was added to the beads, followed by vortexing to fully resuspend them. After standing for 1 min, the supernatant was discarded. The magnetic beads were rinsed twice with 700 μL of 70% ethanol, discarding the supernatant each time, and then dried at 20 °C for 5–10 min to evaporate residual ethanol. Finally, 50 μL of TE buffer (10 mM Tris-HCl and 1 mM EDTA, pH 8.5) was added to release the DNA from the beads by weakening the electrostatic interactions. The mixture was then incubated at 65 °C for 5 min to enhance elution efficiency, and the supernatant was collected and stored at −20 °C.

#### 2.5.4. DNA Extraction Kit

The DNA was extracted in accordance with the instructions provided with the DNA extraction kit.

### 2.6. DNA Quality and Content Testing

#### 2.6.1. Electrophoretic Determination

The genomic DNA extracted using the four methods was analyzed by using a micro-UV spectrophotometer to measure the OD260/280 ratio and DNA concentration. All data were obtained from three independent extractions per sample type (*n* = 3). Statistical analysis was conducted using SPSS version 22.0, and significance was determined using the least significant difference method (*p* < 0.05).

Universal primers for the mitochondrial 16S rRNA gene were designed for PCR amplification, with the primer sequences listed in Table 1. Dilute the upstream and downstream primers to 10 µM before use, and the DNA template was diluted to 1–10^2^ ng/µL. The PCR system comprised 1 μL each of the upstream primer, downstream primer, and DNA template as well as 25 μL of PCR premix. ddH_2_O was added to a final volume of 50 μL. The amplification protocol included an initial denaturation step at 94 °C for 30 s, followed by 30 cycles consisting of denaturation at 94 °C for 30 s, annealing at 55 °C for 30 s, and extension at 72 °C for 1 min, concluding with a final hold at 4 °C.

A total of 5 μL of each PCR product after amplification was mixed with 1 μL of 6× loading buffer. The samples were loaded onto a 1.5% agarose gel and electrophoresed in 1× TAE buffer at a constant voltage of 140 V for 30 min. The DNA bands were visualized and documented using a gel imaging system.

#### 2.6.2. Evaluation of DNA Amplifiability by Real-Time Quantitative PCR (qPCR)

The functional quality of DNA obtained by different extraction methods was assessed using the reference genes and corresponding probes/primers for pig, cattle, sheep, chicken, and duck as specified in regulation of Common detection methods for animal-derived components in livestock and poultry [30], rather than relying solely on purity readings from UV spectrophotometry. All extracted DNA templates were subjected to amplification testing using qPCR. Highly conserved housekeeping genes in eukaryotes were selected as target sequences. DNA amplifiability was further evaluated by comparing the cycle threshold (Ct) values, the linearity of amplification curves, and reproducibility. The qPCR reaction systems are summarized in Table 2. For pig, cattle, sheep, and chicken, reactions were performed under the following conditions: 95 °C for 10 min; 95 °C for 15 s and 60 °C for 1 min, 40 cycles. For duck, the reaction conditions were: 95 °C for 2 min; 95 °C for 15 s and 65 °C for 80 s, 40 cycles.

### 2.7. Anti-Interference Capability Assessment and Analysis of Real Samples

#### 2.7.1. Substrate Interference Test

To test the tolerance of each extraction method to coexisting pollutants, 5% (*w*/*v*) lard (lipids), 2% (*w*/*v*) soluble starch (polysaccharides), and 5% (*w*/*v*) bovine serum albumin (proteins) were individually added to standard DNA solutions of known concentration. DNA recovery efficiency and purity were evaluated by comparing A260/A280 and A260/A230 ratios among samples processed using the different extraction methods. This experimental design enabled a simulation assessment of the anti-interference capability of each method under controlled contamination conditions.

#### 2.7.2. Extraction and Comparative Analysis of Real Samples

To evaluate the extraction performance of four methods in complex matrices, high-calcium milk, whey protein powder, blood sausage, and fresh duck blood were selected as representative samples. Due to the substantial loss of cellular material during processing, whey protein powder contains limited extractable DNA; therefore, the sample input was increased to 300 mg to improve DNA recovery. Three different batches of each sample type were used in the experiment. The practical extraction performance of each method was comprehensively evaluated by comparing DNA yield, purity, and integrity obtained from the real samples. Results are presented as mean ± standard deviation (SD). DNA concentration, A260/A280 ratio, and A260/A230 ratio among the four extraction methods were compared using one-way analysis of variance (ANOVA), followed by Tukey’s honestly significant difference (HSD) post hoc test for pairwise comparisons. The comparison was used to determine whether the four extraction methods produced significant differences in DNA yield and purity within the same matrices of dairy and blood products.

## 3. Results and Discussion

### 3.1. Improve of DNA Extraction Conditions

**Figure 1:**
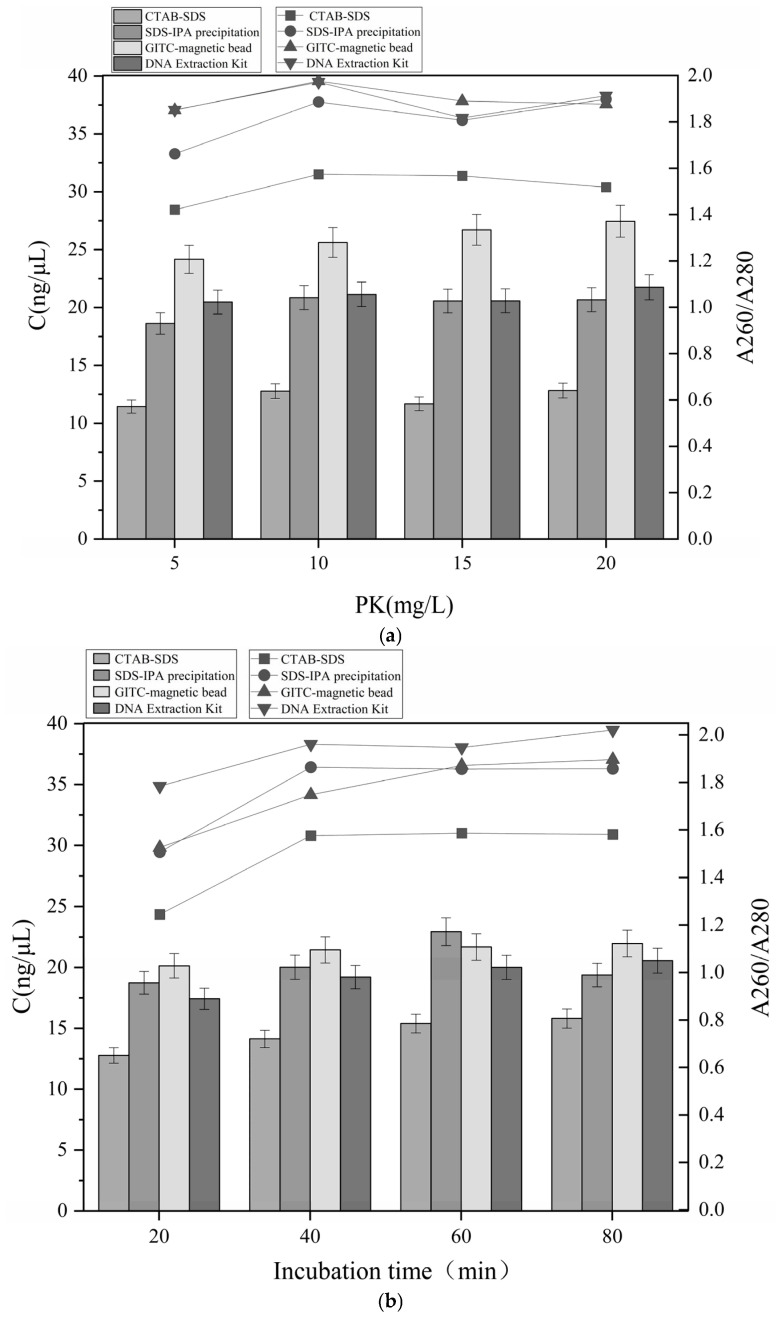
Effects of (**a**) protease K concentration and (**b**) incubation time on DNA extracted from dairy products. The nucleic acid concentration is presented in the column; the A260/A280 ratio is represented by the line.

**Figure 2:**
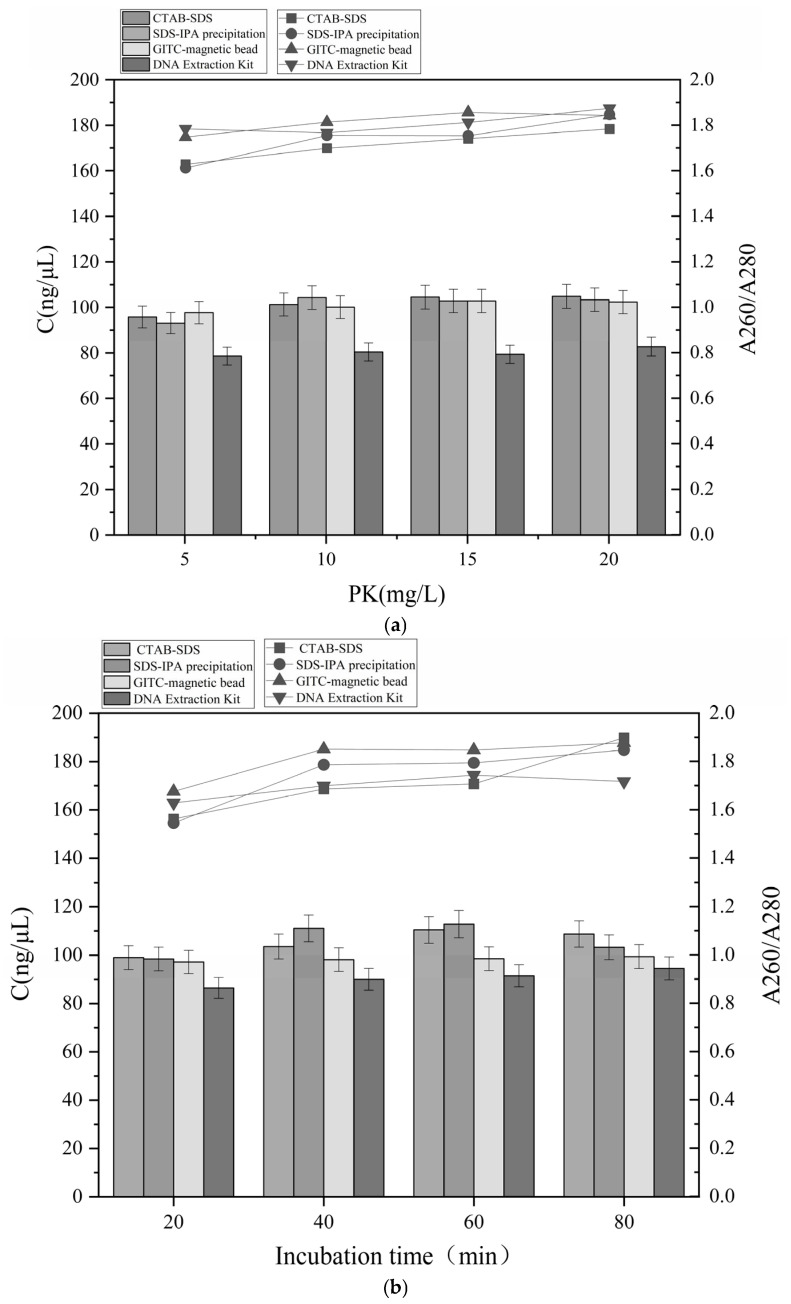
Effects of the (**a**) protease K concentration and (**b**) incubation time on DNA extracted from blood products. The nucleic acid concentration is represented in the column; the A260/A280 ratio is represented by the line.

We systematically evaluated the effects of proteinase K concentration and incubation time on nucleic acid yield and purity to establish an efficient protocol for extracting the DNA from dairy and blood products. The improvement process for dairy products and the corresponding results for the blood-derived matrices was shown in Figure 1 and Figure 2, respectively.

The proteinase K concentration strongly influenced DNA purity, as shown in Figure 1a. The protein was seriously contaminated at 5 mg/mL, substantial protein contamination remained, indicating incomplete protein removal from dairy samples and consequently compromising DNA purity. This was evidenced by the presence of residual proteins; for example, the SDS-CTAB method (A260/A280 = 1.42 ± 0.08), which was consistent with the findings of Qamar et al. [31] for bovine blood. Increasing the proteinase K concentration to 10 mg/mL increased the DNA purity for all extraction methods—except the SDS-CTAB method—with A260/A280 ratios exceeding 1.82 ± 0.05. In addition, the average DNA yield across the four methods increased by 1.43 ng/μL, representing an overall improvement of more than 7%. This result was consistent with the optimal International Dairy Federation conditions for dairy matrices. However, further raising the proteinase K concentration to 15–20 mg/mL, resulted in only marginal improvements in extraction efficiency (approximately 2%). Therefore, increasing the proteinase K concentration beyond 10 mg/mL provided limited additional benefit, likely caused by enzyme saturation [32].

The results of the incubation time improvement were shown in Figure 1b. An incubation time of 60 min, yielded high DNA recovery while DNA purity stabilized. This duration agrees with the 45–75 min range recommended for extracting dairy DNA [33]. Incubation beyond 80 min, the average DNA purity across the four methods declined to 1.79 ± 0.05, and DNA yields also experienced a slight reduction. This decline was likely caused by prolonged incubation, compromising DNA integrity, leading to degradation by nucleases.

Similar trends emerged, although with matrix-specific variations, in the results of blood product analysis, which were presented in Figure 2. At a proteinase K concentration of 10 mg/mL, the DNA yield obtained using the four methods increased by an average of 5.21 ng/μL compared with the preceding concentration level. Further increases to 15 and 20 mg/mL, resulted in smaller gains of 1.13 ng/μL and 0.925 ng/μL, respectively, while improvements in DNA purity also plateaued.

A comparable pattern was observed for incubation time. DNA purity stabilized and yield increased at 60 min; however, extending the incubation time to 80 min led to a decline in yield. These differences highlight the need to improve the conditions for each matrix, as previously discussed [34].

Following condition improvement, the final protocol employed 10 mg/mL proteinase K with a total incubation time of approximately 60 min, which was consistently applied in subsequent experiments involving dairy and blood products.

### 3.2. DNA Extraction Results of Four Extraction Methods

We systematically compared the SDS-CTAB method, SDS-isopropanol precipitation, guanidine isothiocyanate-magnetic bead separation, and a commercial DNA extraction kit to assess the differences in DNA extraction from dairy- and blood-based food matrices. The DNA yield and purity (A260/A280) results were summarized in Table 3.

The DNA yield obtained using the SDS-CTAB method showed the most variability. Although relatively high DNA concentrations were achieved in certain samples, such as milk powder and duck blood, the A260/A280 ratios were frequently low—for example, approximately 1.24 in milk slice and 1.35 in goat milk powder. This method appears less reliable when applied to complex or processed matrices. These values align with earlier findings that the CTAB-based method has limitations when used with complex food matrix samples with high protein and lipid contents [20].

The purity of the DNA obtained with the SDS-isopropanol precipitation method was higher than that obtained with the SDS-CTAB method. The A260/A280 ratios ranged from 1.7 to 1.8 for the dairy products and exceeded 1.8 for the blood samples, indicating the protein impurities were more effectively removed than with the SDS-CTAB method. In addition, this method generally produced relatively high DNA yields and ranked among the top-performing approaches in several matrices (e.g., milk, yogurt, and goat milk powder). However, its purity remained slightly variable, with A260/A280 ratios typically between 1.7 and 1.8, suggesting minor protein carryover in some cases. This finding supports those of Kurita et al. [35], who found that isopropanol precipitation reduced the protein and polysaccharide contamination of viscous samples.

Among the evaluated protocols, the guanidinium isothiocyanate–magnetic bead method demonstrated the most consistent purity. The A260/A280 ratios from dairy and blood were 1.7–1.8 and ≥1.9, respectively with minimal variability (coefficient of variation (CV) ≤ 3.5%). In addition, this method produced A260/A230 ratios close to or above 2.0 (1.95–1.98 for dairy products and 2.02–2.15 for blood), reflecting highly efficient removal of salts, carbohydrates, and chaotropic reagents. This suggests effective removal of both proteins and inhibitors. However, yield varies depending on the sample matrix and was sometimes lower in highly processed samples, and purer than that obtained with DNA extraction kit silica column methods [36]. This method also does not require phenol and chloroform, in line with the EFSA green chemistry principles [37].

The commercial DNA extraction kit employs a modified CTAB lysis protocol and eliminates the need for phenol-chloroform extraction. It relies on the selective adsorption properties of a silica membrane spin column to efficiently remove proteins and other contaminants. The commercial DNA extraction kit consistently produced pure DNA (A260/A280 > 1.8) and A260/A230 ratios close to 2.0 across all sample types, indicating effective removal of both protein and non-protein contaminants. However, DNA yields obtained with the kit were 20–30% lower than those achieved using the magnetic bead method for milk, milk powder, duck blood, and other dairy and blood products. In some cases (e.g., yogurt, milk powder), were also lower than those achieved with conventional chemical extraction methods. Despite this, the method demonstrated good reproducibility, as evidenced by relatively low standard deviations. This reflects the tradeoff between purity and yield, as noted by manufacturers and cost-efficiency studies [20].

Among the eight substrate samples, triplicate tests on the same batch showed that, for liquid dairy products, the magnetic bead method achieved high yields with good purity, while the kit-based method produced cleaner DNA at lower yield; the SDS-isopropanol method provided comparable yield but slightly reduced purity. In processed dairy matrices, these differences were more pronounced: SDS-CTAB yielded the highest concentrations but showed lower A260/A280 ratios, indicating residual contamination, whereas magnetic bead and kit-based methods produced higher-purity DNA at lower yields with stable A260/A230 ratios suitable for PCR. In goat milk powder, SDS-isopropanol gave the highest yield, while magnetic bead extraction provided superior purity, and SDS-CTAB showed limited protein removal. In blood samples, all methods generated high yields; however, magnetic bead and kit-based methods consistently produced higher-purity DNA, SDS-isopropanol maintained a balance between yield and purity, and SDS-CTAB remained less effective in removing protein contaminants.

Incubation time varied substantially among the methods (Table 4). The magnetic bead method required only 20 min for incubation, with subsequent DNA separation, purification, and elution completed within an additional 18 min, resulting in the shortest overall processing time. In comparison, the commercial kit required approximately 100 min, whereas the SDS-CTAB and SDS-isopropanol methods required 140 min or more. Overall, the magnetic bead method reduced processing time by approximately 40% relative to the kit-based approach.

The costs of preparing reagents in the laboratory for SDS-CTAB, SDS-IPA precipitation, and GITC-magnetic bead methods were approximately 0.6 USD, 0.3 USD, and 0.9 USD per extraction, respectively. In comparison, the commercial DNA extraction kit costs around 180 USD and can be used for 50 extractions, resulting in an average cost of 3.6 USD per extraction. Among the laboratory-prepared methods, SDS-CTAB and SDS-IPA precipitation was relatively inexpensive, while the GITC-magnetic bead method incurs a slightly higher cost but remains substantially lower than that of the commercial kit.

### 3.3. Evaluation of DNA Amplifiability of DNA Extracted by Four Methods Using Electrophoresis and qPCR

**Figure 3:**
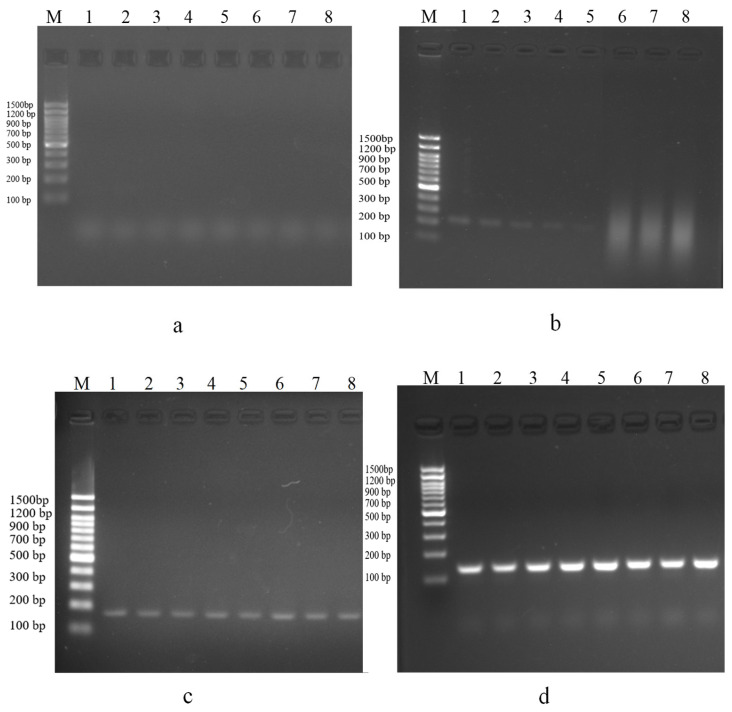
Comparison of electrophoresis results of four extraction methods. (**a**–**d**) SDS-CTAB, SDS-IPA precipitation, GITC-magnetic bead, and DNA extraction kit methods. M, marker 1500 bp ladder; 1 to 8 represent milk, yogurt, milk slice, goat milk powder, milk powder, duck blood, chicken blood, and pig blood, respectively.

**Figure 4:**
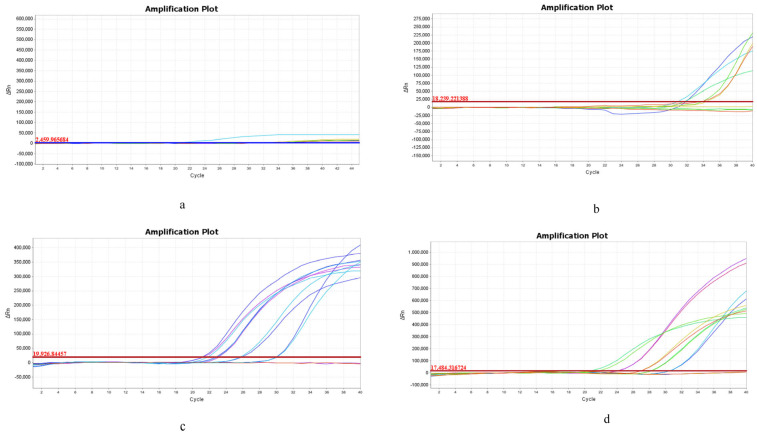
Comparison of qPCR results of four extraction methods. (**a**–**d**) SDS-CTAB, SDS-IPA precipitation, GITC-magnetic bead, and DNA extraction kit methods.

The results of the downstream analyses were shown in Figure 3. The DNA obtained from the CTAB extracts produced faint or absent bands, indicating protein contamination. In contrast, the DNA purified with magnetic beads produced clear, reproducible bands across all samples, outperforming the SDS-isopropanol precipitation method, which produced variable band intensities that matched those of the commercial kit. This finding supports that of a prior study showing that silica-based purification effectively removed PCR inhibitors [38]. qPCR results (Figure 4) were largely consistent with electrophoresis findings. The SDS-CTAB method failed to produce detectable amplification curves, indicating substantial PCR inhibition. For SDS-IPA, amplification was mostly absent, with dairy samples showing curves only near a Ct of 32, accompanied by low fluorescence intensity and poor reproducibility. The magnetic bead method generated amplification curves for all samples with Ct values of 21–30, although fluorescence intensity varied among replicates. The commercial kit produced curves in the same Ct range with higher fluorescence intensity and minimal replicate variation. While the magnetic bead method showed slightly lower fluorescence and reproducibility than the kit, its stability can be improved through further optimization of the extraction reagents to approach kit-level performance.

The magnetic bead method was a cost-effective and eco-friendly alternative for authenticating food products, as demonstrated by its high DNA yield (e.g., 318.34 ± 4.77 ng/µL from chicken blood), high DNA purity (1.89 ± 0.07), and absence of toxic solvents. This method addresses the challenge of co-extracting contaminants from substrates that are difficult to process, particularly for testing processed foods in which DNA was often degraded, which was a concern raised by the EU Food Fraud Network [39].

### 3.4. Results of Substrate Interference and Actual Sample Analysis

As shown in Table 5, the guanidinium isothiocyanate-magnetic bead method was evaluated for its tolerance to coexisting contaminants and exhibited stable resistance to potential interfering substances. Analysis of variance indicated significant differences between the GITC−magnetic bead method and the SDS−IPA and SDS−CTAB methods, with the GITC−magnetic bead method exhibiting higher extraction efficiency. Among the three methods, the magnetic bead method achieved the highest DNA content, and its yield and purity were comparable to those of the commercial kit. Notably, under polysaccharide contamination, the extraction efficiency of the GITC−magnetic bead method was comparable to that of the commercial DNA extraction kit. Similar robustness of magnetic bead−based extraction systems in complex food and biological matrices has been reported previously, which has been attributed to their strong denaturing capacity and selective DNA binding efficiency [20,40]. However, this evaluation was limited to contaminant tolerance in the presence of DNA and does not assess extraction performance from natural food matrices. Therefore, four types of natural or processed dairy and blood products, representing different extraction difficulties, were selected for the subsequent food matrix study.

Three samples from different batches were selected as replicates to assess the method’s adaptability to samples from various sources. For complex dairy and blood−derived products (Table 6), clear method−dependent differences were observed. In calcium−fortified milk, DNA yields were comparable to those of conventional samples; however, lower purity values obtained with the SDS−CTAB and SDS−IPA methods suggest limited removal of co−extracted impurities. This observation is consistent with previous reports indicating that CTAB−based protocols are less effective for matrices with high fat and protein contents, owing to the co-extraction of interfering components [41].

Whey protein powder, characterized by high protein content and heat-induced DNA degradation, showed generally low extraction efficiency. Among the tested methods, SDS-IPA yielded the lowest recovery, likely due to insufficient disruption of denatured protein matrices. Similarly, blood sausage, which contains starch and undergoes thermal processing, exhibited reduced DNA yields [42]. Overall, compared with other methods, the magnetic bead-based method demonstrated greater stability and higher yields in highly processed samples and was less affected by matrix-related interferences. Collectively, these results indicate that the evaluated DNA extraction methods showed good reproducibility across different matrices.

## 4. Conclusions

This study addressed the challenges faced in food authenticity testing through evaluating four methods for extracting DNA from dairy and blood products. The guanidine isothiocyanate magnetic bead method outperformed the other considered techniques, including SDS-CTAB and SDS-isopropanol. Overall, the magnetic bead method outperformed the SDS-CTAB and SDS-IPA precipitation methods in the tested dairy and blood products and exhibited performance comparable to that of commercial kits. However, its efficacy in other food matrices has not been assessed, and it remains unclear whether it would match the performance of commercial kits in those contexts. This magnetic bead-based approach yields DNA of high concentration and purity (A260/A280 = 1.8–2.0) and, unlike methods such as the CTAB technique, does not require toxic phenol and chloroform reagents, in line with green chemistry principles. In addition, the absence of centrifugation steps and the simplified workflow make this method particularly suitable for complex matrices such as blood and dairy products, where it effectively reduces lipid interference and removes heme-derived inhibitors. Future studies should functionally modify magnetic beads and buffer systems to further increase their sensitivity and applicability to a wider range of food matrices. Moreover, application to challenging samples, including highly processed foods and environmental matrices such as soil, may further demonstrate its potential for efficient recovery of high-quality DNA suitable for downstream molecular analyses.

## Figures and Tables

**Table 1:** Primers and sequences used for DNA PCR amplification.

Gene	Primer Sequence (5′ → 3′)	Fragment Length
Forward primer	TTACGACCTCGATGTTGGAT	128 bp
Reverse primer	ACAGATAGAAACCGACCTGG	

**Table 2:** Real-time PCR system.

Ingredient	Concentration of Working Fluid	Volume (μL)
DNA	5–10 ng/μL	1.0
qPCR Premixed night	2×	10.0
forward primer	10 μM	0.4
reverse primer	10 μM	0.4
probe	10 μM	0.4
ROX-II	50×	0.2
ddH_2_O	/	7.6
Total		20

**Table 3:** Comparison of DNA extraction performance across four methods (mean ± SD).

Sample	Method	C (ng/μL)	A260/A280	A260/A230
Milk	SDS-CTAB	12.12 ± 0.74	1.452 ± 0.041	1.728 ± 0.029
	SDS-IPA precipitation	18.69 ± 0.50	1.765 ± 0.032	1.798 ± 0.068
	GITC-Magnetic bead	27.85 ± 0.80	1.789 ± 0.050	1.950 ± 0.049
	DNA Extraction Kit	20.85 ± 0.63	1.862 ± 0.024	1.961 ± 0.027
Yogurt	SDS-CTAB	117.65 ± 1.50	1.575 ± 0.026	1.763 ± 0.027
	SDS-IPA precipitation	128.23 ± 0.53	1.823 ± 0.028	1.741 ± 0.049
	GITC-Magnetic bead	136.76 ± 0.89	1.828 ± 0.020	1.960 ± 0.034
	DNA Extraction Kit	87.96 ± 1.06	1.824 ± 0.045	1.971 ± 0.055
Milk slice	SDS-CTAB	133.31 ± 1.81	1.241 ± 0.023	1.791 ± 0.043
	SDS-IPA precipitation	121.94 ± 2.49	1.736 ± 0.072	1.826 ± 0.043
	GITC-Magnetic bead	47.97 ± 1.48	1.789 ± 0.029	1.895 ± 0.022
	DNA Extraction Kit	43.45 ± 0.58	1.827 ± 0.036	1.954 ± 0.020
Goat milk powder	SDS-CTAB	64.11 ± 2.56	1.358 ± 0.061	1.846 ± 0.010
	SDS-IPA precipitation	121.44 ± 1.73	1.646 ± 0.032	1.774 ± 0.065
	GITC-Magnetic bead	64.73 ± 0.55	1.800 ± 0.077	1.954 ± 0.029
	DNA Extraction Kit	57.01 ± 1.60	1.784 ± 0.024	1.990 ± 0.016
Milk powder	SDS-CTAB	100.29 ± 1.69	1.533 ± 0.039	1.779 ± 0.064
	SDS-IPA precipitation	47.85 ± 2.03	1.740 ± 0.026	1.817 ± 0.058
	GITC-Magnetic bead	38.32 ± 1.16	1.740 ± 0.049	1.982 ± 0.060
	DNA Extraction Kit	27.84 ± 1.11	1.845 ± 0.018	1.918 ± 0.040
Duck blood	SDS-CTAB	279.53 ± 4.61	1.672 ± 0.020	1.888 ± 0.026
	SDS-IPA precipitation	270.50 ± 7.05	1.860 ± 0.017	1.918 ± 0.038
	GITC-Magnetic bead	259.90 ± 4.73	1.935 ± 0.059	2.015 ± 0.119
	DNA Extraction Kit	275.37 ± 3.20	1.939 ± 0.036	2.036 ± 0.066
Chicken blood	SDS-CTAB	299.50 ± 2.23	1.774 ± 0.053	1.895 ± 0.033
	SDS-IPA precipitation	301.82 ± 4.11	1.838 ± 0.038	1.838 ± 0.039
	GITC-Magnetic bead	318.34 ± 4.77	1.886 ± 0.066	2.027 ± 0.105
	DNA Extraction Kit	252.05 ± 3.77	1.912 ± 0.032	2.029 ± 0.063
Pig blood	SDS-CTAB	100.01 ± 4.33	1.583 ± 0.026	1.895 ± 0.028
	SDS-IPA precipitation	120.31 ± 2.85	1.815 ± 0.055	1.938 ± 0.013
	GITC-Magnetic bead	84.67 ± 4.98	2.022 ± 0.125	2.021 ± 0.061
	DNA Extraction Kit	72.39 ± 3.74	1.916 ± 0.051	2.003 ± 0.059

Values are presented as mean ± standard deviation (*n* = 3).

**Table 4:** The time required for each DNA extraction method.

Method	Incubation Time	Total Centrifugation Time	DNA Separation, Purification and Drying	Estimated Time	Actual Duration
SDS-CTAB	75 min	40 min	60 min	179 min	200 min
SDS-IPA precipitation	60 min	13 min	55 min	128 min	140 min
GITC-Magnetic bead	20 min	/	18 min	38 min	60 min
DNA Extraction Kit	60 min	14 min	16 min	90 min	100 min

**Table 5:** Substrate interference test results.

Interference	Method	Mean Value (ng/μL)	Group			
Lipid	SDS−CTAB	78.87 ± 1.66			C	
	SDS−IPA precipitation	75.14 ± 1.39				D
	GITC−Magnetic bead	85.39 ± 0.71		B		
	DNA Extraction Kit	91.96 ± 1.54	A			
Polysaccharide	SDS−CTAB	72.55 ± 1.07				D
	SDS−IPA precipitation	86.60 ±1.94			C	
	GITC−Magnetic bead	89.88 ± 1.06		B		
	DNA Extraction Kit	90.17 ± 0.42	A			
Protein	SDS−CTAB	82.49 ± 1.48			C	
	SDS−IPA precipitation	76.53 ± 0.77				D
	GITC−Magnetic bead	86.28 ± 1.53		B		
	DNA Extraction Kit	92.37 ± 1.58	A			

Values are presented as mean ± standard deviation (*n* = 3), and the methods of not sharing letters are significantly different (*p* < 0.05).

**Table 6:** Actual sample testing and analysis results.

Sample	Number	Extraction Method	$\bar{x}$ ± SD (ng/µL)	A260/A280 ± SD	A260/A230 ± SD
high-calcium milk	3	SDS-IPA precipitation	20.21 ± 0.44	1.644 ± 0.05	1.684 ± 0.057
	3	SDS-CTAB	14.12 ± 0.51	1.482 ± 0.042	1.621 ± 0.022
	3	GITC-Magnetic bead	27.54 ± 0.74	1.820 ± 0.050	1.964 ± 0.056
	3	DNA Extraction Kit	20.41 ± 0.55	1.855 ± 0.021	1.978 ± 0.019
whey protein powder	3	SDS-IPA precipitation	2.31 ± 0.38	1.648 ± 0.035	1.705 ± 0.068
	3	SDS-CTAB	7.62 ± 0.12	1.521 ± 0.047	1.756 ± 0.051
	3	GITC-Magnetic bead	11.44 ± 0.61	1.814 ± 0.048	1.961 ± 0.012
	3	DNA Extraction Kit	10.25 ± 0.57	1.901 ± 0.019	1.957 ± 0.017
blood sausage	3	SDS-IPA precipitation	8.27 ± 0.38	1.678 ± 0.039	1.698 ± 0.064
	3	SDS-CTAB	6.39 ± 0.22	1.527 ± 0.057	1.531 ± 0.055
	3	GITC-Magnetic bead	12.70 ± 0.64	1.866 ± 0.026	1.916 ± 0.022
	3	DNA Extraction Kit	11.16 ± 0.49	1.921 ± 0.011	1.957 ± 0.020
fresh duck blood	3	SDS-IPA precipitation	123.02 ± 2.47	1.786 ± 0.052	1.857 ± 0.037
	3	SDS-CTAB	98.47 ± 5.02	1.578 ± 0.041	1.852 ± 0.026
	3	GITC-Magnetic bead	88.27 ± 3.25	1.952 ± 0.064	2.032 ± 0.065
	3	DNA Extraction Kit	75.52 ± 3.84	1.927 ± 0.051	2.024 ± 0.051

Values are presented as mean ± standard deviation (*n* = 3).

## Data Availability

The original contributions presented in this study are included in the article. Further inquiries can be directed to the corresponding authors.

## References

[1] Singh R., Puniya A.K. (2024). Role of food safety regulations in protecting public health. Indian J. Microbiol..

[2] Barennes H., Empis G., Quang T.D., Sengkhamyong K., Phasavath P., Harimanana A., Sambany E.M., Koffi P.N. (2012). Breast-milk substitutes: A new old-threat for breastfeeding policy in developing countries. A case study in a traditionally high breastfeeding country. PLoS ONE.

[3] Dror D.K., Allen L.H. (2014). Dairy product intake in children and adolescents in developed countries: Trends, nutritional contribution, and a review of association with health outcomes. Nutr. Rev..

[4] Henchion M., Moloney A., Hyland J., Zimmermann J., McCarthy S.J.A. (2021). Trends for meat, milk and egg consumption for the next decades and the role played by livestock systems in the global production of proteins. Animal.

[5] Yang S., Bhargava N., O’Connor A., Gibney E.R., Feeney E.L. (2023). Dairy consumption in adults in China: A systematic review. BMC Nutr..

[6] Kurhaluk N., Gradziuk M., Tkaczenko H. (2024). Optimisation of Blood Donor Nutrition: Blood Donor Health Improvement Studies. Cell. Physiol. Biochem..

[7] Fang J., Xing J., Xu X., Mao L., Zhu H., Wu Y., Cheng H., Chen C., Shi L., Yang Z. (2024). Research progress on the authenticity of duck blood. Microchem. J..

[8] DiMarket (2024). Duck Blood Future-Proof Strategies: Market Trends 2025–2033.

[9] Esteki M., Regueiro J., Simal-Gándara J. (2019). Tackling fraudsters with global strategies to expose fraud in the food chain. Compr. Rev. Food Sci. Food Saf..

[10] Gwenzi W., Makuvara Z., Marumure J., Simbanegavi T.T., Mukonza S.S., Chaukura N. (2023). Chicanery in the food supply chain! Food fraud, mitigation, and research needs in low-income countries. Trends Food Sci. Technol..

[11] Cheng C., Chen L., Zhang D., Yu J., Zhu M., Li C., Zheng X., Blecker C., Li S. (2024). Value-added utilization of hemoglobin and its hydrolysis products from livestock and poultry blood processing by-products: A review. Trends Food Sci. Technol..

[12] Kamal M., Karoui R. (2015). Analytical methods coupled with chemometric tools for determining the authenticity and detecting the adulteration of dairy products: A review. Food Chem..

[13] Ye H., Yang J., Xiao G., Zhao Y., Li Z., Bai W., Zeng X., Dong H. (2023). A comprehensive overview of emerging techniques and chemometrics for authenticity and traceability of animal-derived food. Food Chem..

[14] Vishnuraj M., Kumar N.A., Vaithiyanathan S., Barbuddhe S.B. (2023). Authentication issues in foods of animal origin and advanced molecular techniques for identification and vulnerability assessment. Trends Food Sci. Technol..

[15] Matsuda K. (2017). PCR-based detection methods for single-nucleotide polymorphism or mutation: Real-time PCR and its substantial contribution toward technological refinement. Adv. Clin. Chem..

[16] Cha R.S., Thilly W.G. (1993). Specificity, efficiency, and fidelity of PCR. Genome Res..

[17] Schrader C., Schielke A., Ellerbroek L., Johne R. (2012). PCR inhibitors-occurrence, properties and removal. J. Appl. Microbiol..

[18] Pereira F., Carneiro J., Amorim A. (2008). Identification of species with DNA-based technology: Current progress and challenges. Recent Pat. DNA Gene Seq..

[19] Qian J., Dai B., Wang B., Zha Y., Song Q. (2022). Traceability in food processing: Problems, methods, and performance evaluations—A review. Crit. Rev. Food Sci. Nutr..

[20] Sajali N., Wong S.C., Hanapi U.K., Abu Bakar Jamaluddin S., Tasrip N.A., Mohd Desa M.N. (2018). The challenges of DNA extraction in different assorted food matrices: A review. J. Food Sci..

[21] Liao J., Liu Y., Yang L., Li F., Sheppard A.M. (2017). Development of a rapid mitochondrial DNA extraction method for species identification in milk and milk products. J. Dairy Sci..

[22] Quigley L., O’Sullivan O., Beresford T., Paul Ross R., Fitzgerald G., Cotter P.D. (2012). A comparison of methods used to extract bacterial DNA from raw milk and raw milk cheese. J. Appl. Microbiol..

[23] Arslan M. (2022). Effects of centrifugation and washing of freeze-thawed blood on isolated DNA characteristics. Turk. J. Vet. Anim. Sci..

[24] Huang X., Duan N., Xu H., Xie T., Xue Y.-R., Liu C.-H. (2018). CTAB-PEG DNA extraction from fungi with high contents of polysaccharides. Mol. Biol..

[25] Nwokeoji A.O., Kilby P.M., Portwood D.E., Dickman M.J. (2016). RNASwift: A rapid, versatile RNA extraction method free from phenol and chloroform. Anal. Biochem..

[26] Xia Y., Chen F., Du Y., Liu C., Bu G., Xin Y., Liu B. (2019). A modified SDS-based DNA extraction method from raw soybean. Biosci. Rep..

[27] Xin S., Hongzhi C., Baozhong H., Sandui G. (2004). A SDS-CTAB combined method for extracting total DNA from cotton plant. Biotechnol. Bull..

[28] Liang M., Xiao J., Chen K., Huang Z., Lao J. (2021). Extraction of plant protein beverage DNA by magnetic beads. J. Food Saf. Qual..

[29] Lustig G., Ganga Y., Rodel H.E., Tegally H., Khairallah A., Jackson L., Cele S., Khan K., Jule Z., Reedoy K. (2024). SARS-CoV-2 infection in immunosuppression evolves sub-lineages which independently accumulate neutralization escape mutations. Virus Evol..

[30] (2019). Biochemistry Products and Testing Technology (SAC/TC 387). Identification of Animal Ingredient from Common Livestock and Poultry—Real-Time PCR.

[31] Qamar W., Khan M.R., Arafah A. (2017). Optimization of conditions to extract high quality DNA for PCR analysis from whole blood using SDS-proteinase K method. Saudi J. Biol. Sci..

[32] Fox P.F., McSweeney P.L. (1998). Dairy Chemistry and Biochemistry.

[33] Mertens K., Freund L., Schmoock G., Hänsel C., Melzer F., Elschner M.C. (2014). Comparative evaluation of eleven commercial DNA extraction kits for real-time PCR detection of *Bacillus anthracis* spores in spiked dairy samples. Int. J. Food Microbiol..

[34] Unger A.L., Astrup A., Feeney E.L., Holscher H.D., Gerstein D.E., Torres-Gonzalez M., Brown K. (2023). Harnessing the Magic of the Dairy Matrix for Next-Level Health Solutions: A Summary of a Symposium Presented at Nutrition 2022. Curr. Dev. Nutr..

[35] Kurita O., Murakami K., Fujiwara T. (2010). Chemical modification of polysaccharides by the use of intramolecular associations in polar organic solvents. Polym. Bull..

[36] Ye X., Lei B. (2023). The current status and trends of DNA extraction. BioEssays.

[37] European Food Safety Authority (2023). Commission Regulation (EU) 2023/915 of 25 April 2023 on Maximum Levels for Certain Contaminants in Food. https://eur-lex.europa.eu/eli/reg/2023/915/oj.

[38] Mirna Lorena S., Cynthia P.C., Maria Isabela A.R., Pamela V.M., Gabriela R.H., Jorge B., Mariano G. (2023). Nucleic acids isolation for molecular diagnostics: Present and future of the silica-based DNA/RNA purification technologies. Sep. Purif. Rev..

[39] Montanari F., Varallo C., Pisanello D. (2016). Food Fraud in the EU. Eur. J. Risk Regul..

[40] Dester E., Alocilja E. (2022). Current methods for exstraction and concentration of foodborne bacteria with glycan-coated magnetic nanoparticles: A review. Biosensors.

[41] Roopnarain A., Mukhuba M., Adeleke R., Moeletsi M. (2017). Biases during DNA extraction affect bacterial and archaeal community profile of anaerobic digestion samples. 3 Biotech.

[42] Esa M.S., Faujan N.H., Rajab H.A., Rehan M.M., Arifin N., Rahim A.A. (2018). Comparison of DNA Concentration and Purity of Animal Blood Extracted Using Different DNA Extraction Kits. Malays. J. Sci. Health Technol..

